# Supplementary material for: Do Activity Sensors Identify Physiological, Clinical and Behavioural Changes in Laying Hens Exposed to a Vaccine Challenge?
Source: Animals (Basel). 2025 Jan 14;15(2):205. doi: 10.3390/ani15020205 (PMC11758295; doi:10.3390/ani15020205)
Supplement: Supplementary file 1 [file animals-15-00205-s001.zip › Figure S1.pdf]

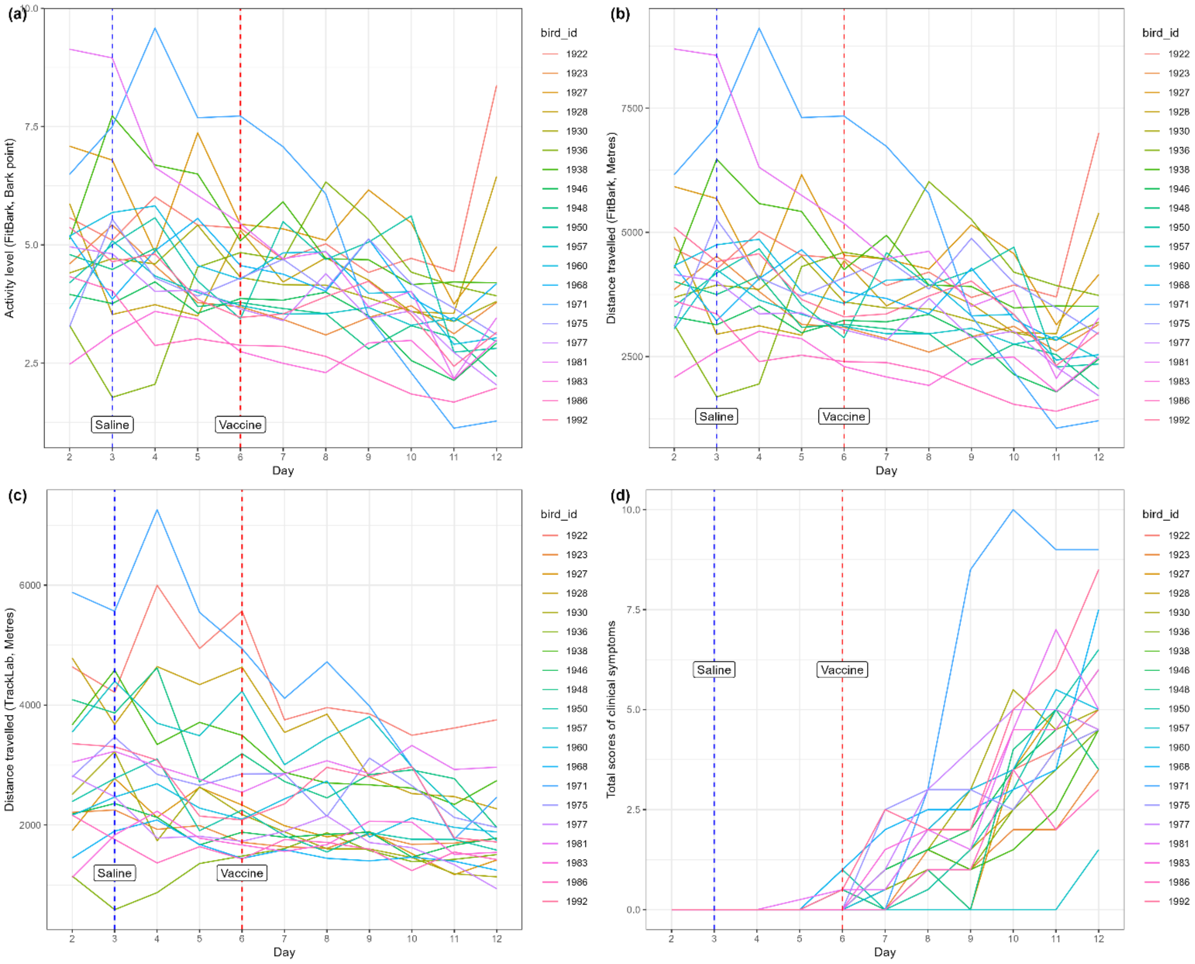

Figure S1. Daily data values for each hen of (a) activity level by FitBark, (b) distance travelled by FitBark, (c) distance travelled by TrackLab, (d) total scores of clinical symptoms. The days on which saline and a live vaccine were administered to birds are indicated. Bird_ids 1971, 1981, 1975, 1936, 1992 were wearing FitBark tag 1, for which the relationship between activity level and distance travelled differed slightly from the relationship for the other 3 tags (see Figure S2).
