# Supplementary material for: Do Activity Sensors Identify Physiological, Clinical and Behavioural Changes in Laying Hens Exposed to a Vaccine Challenge?
Source: Animals (Basel). 2025 Jan 14;15(2):205. doi: 10.3390/ani15020205 (PMC11758295; doi:10.3390/ani15020205)
Supplement: Supplementary file 1 [file animals-15-00205-s001.zip › Figure S2.pdf]

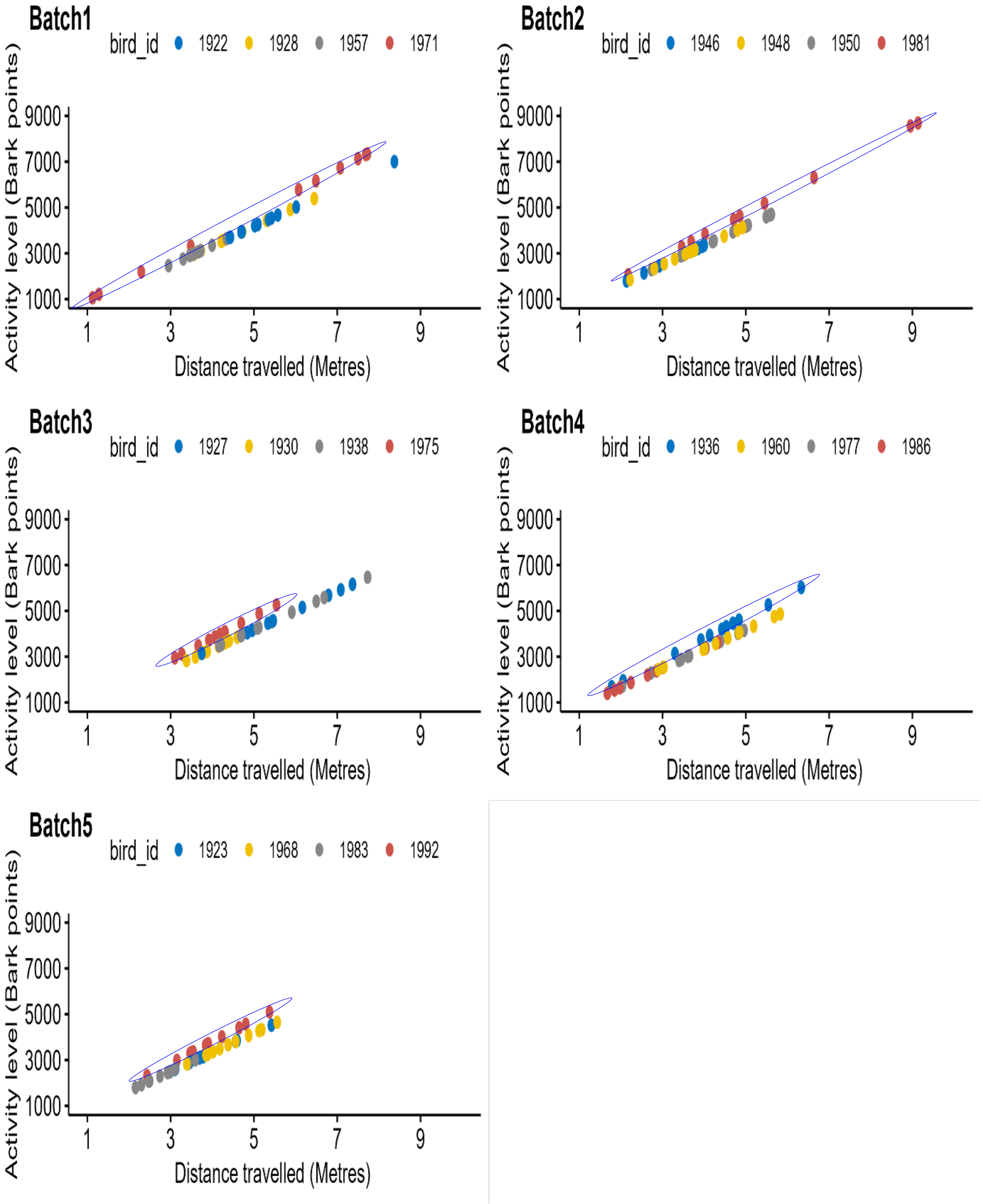

Figure S2. Scatter plots of distance travelled and activity level by FitBark per batch. Blue circles indicate birds that were wearing tag 1. The relationship between activity level and distance travelled is identical for tag 2, 3 and 4 for all four batches, and identical for tag 1 for all four batches, but is slightly different for tag 1 than it is for tags 2, 3 and 4.
